# Circ_0013359 facilitates the tumorigenicity of melanoma by regulating miR-136-5p/RAB9A axis

**DOI:** 10.1515/biol-2021-0030

**Published:** 2021-05-22

**Authors:** Qi Zhang, Yingfa Feng, Jiangang Feng, Jinming Zhang, Lili Huang

**Affiliations:** Department of Ultrasound, Hebei Chest Hospital, Shijiazhuang, Hebei, China; Department of Orthopedics, The Fourth Hospital of Hebei Medical University, No. 12, Jiankang Road, Shijiazhuang, 050011 Hebei, China; Department of Orthopedics, Weichang Hospital of Traditional Chinese Medicine, Chengde, Hebei, China

**Keywords:** melanoma, circ_0013359, miR-136-5p, RAB9A, glycolysis

## Abstract

**Background:**

Circular RNAs play crucial roles in tumor occurrence and progression. This research aimed to explore the role and potential mechanism of hsa_circ_0013359 (circ_0013359) in melanoma.

**Methods:**

The levels of circ_0013359, microRNA-136-5p (miR-136-5p), and member RAS oncogene family (RAB9A) in melanoma tissues and cells were detected using quantitative reverse transcriptase-polymerase chain reaction or western blot. Cell proliferation, apoptosis, cell cycle, cell migration, and invasion were evaluated by 3-(4,5-dimethyl-2-thiazolyl)-2,5-diphenyl-2-*H*-tetrazolium bromide assay, colony formation assay, flow cytometry, and transwell assay. Glycolysis was determined by detecting glucose consumption, lactate production, and extracellular acidification rate. The levels of hexokinase 2 and lactate dehydrogenase A were examined by western blot. The targeting relationship between miR-136-5p and circ_0013359 or RAB9A was confirmed by dual-luciferase reporter assay. Xenograft experiments were used to analyze tumor growth *in vivo*.

**Results:**

Circ_0013359 and RAB9A levels were increased, while the miR-136-5p level was reduced in melanoma tissues and cells. Circ_0013359 knockdown inhibited proliferation, migration, invasion, and glycolysis and promoted apoptosis and cycle arrest in A875 and SK-MEL-1 cells. Circ_0013359 sponged miR-136-5p to regulate melanoma progression. In addition, miR-136-5p suppressed melanoma progression by targeting RAB9A. Besides, circ_0013359 silencing inhibited tumor growth *in vivo*.

**Conclusion:**

Depletion of circ_0013359 hindered melanoma progression by regulating miR-136-5p/RAB9A axis.

## 1 Introduction

Melanoma is an aggressive skin cancer and the leading cause of skin cancer-related death [1]. Recently, the incidence of melanoma has increased year by year, while metastasis is still a serious obstacle to melanoma treatment [2,3]. Melanoma originates from melanocytes, accounting for only 5% of all skin cancers [4]. Among them, the 5-year survival rate of metastatic melanoma patients is only 5% [5]. Human adipose-derived mesenchymal stem cells expressing IP-10 could reduce melanoma tumor growth and lung metastasis [6]. Therefore, further understanding of melanoma’s underlying mechanisms is urgently needed to improve the poor prognosis of melanoma.

Circular RNAs (circRNAs) possess unique closed-loop structures without 5′ to 3′ polarity and are widely distributed in various eukaryotes [7]. Mounting evidence has demonstrated that circRNAs participate in multiple biological processes and play crucial roles in diverse cancers [8]. Besides, many aberrantly expressed circRNAs can serve as new diagnostic markers in various skin diseases, including melanoma [9]. For example, circRNA_0084043 facilitated melanoma progression by inducing tumor cell proliferation and motility [10]. Moreover, hsa_circ_0025039 enhanced the malignant behaviors of melanoma via promoting tumor cell growth, invasion, and glucose [11]. Besides, circular RNA itchy E3 ubiquitin protein ligase decelerated melanoma cell proliferation by repressing glucose uptake [12]. In addition, hsa_circ_0013359 derived from collagen type XI alpha 1 chain (COL11A1) is located on chr1:103477968-103487325. A previous study showed that circ_0013359 was strikingly upregulated in malignant melanoma through microarrays [10]. Nonetheless, the exact role and possible mechanism of circ_0013359 in melanoma remain unclear.

Emerging evidence has verified that circRNAs function as microRNA (miRNA) sponges, competing for miRNA binding sites and indirectly regulating gene expression [13]. Moreover, numerous investigations have suggested that miRNAs are dysregulated in melanoma and are intimately related to melanoma development, so miRNAs are potential diagnostic and prognostic biomarkers for melanoma [14,15]. For instance, Zhang and Yang unveiled that miR-33a-5p hampered melanoma cell growth and accelerated apoptosis via interacting with SNAI2 [16]. Yang et al. claimed that elevation of miR-489-3p suppressed melanoma cell development and glycolysis by binding to SIX1 [17]. He et al. showed that miR-140-3p reduced the malignant phenotype of cutaneous melanoma by targeting ABHD2 to regulate JNK and AKT/p70S6K pathways [18]. Besides, miRNA-136 was overtly downregulated in melanoma and its elevation inhibited tumor progression via repression of PMEL and inactivation of the Wnt signaling pathway [19]. However, the association between circ_0013359 and miR-136-5p has not been investigated. In addition, member RAS oncogene family (RAB9A) is classified as the Rab GTPase family, which are essential regulators of membrane traffic [20]. Previous research unveiled that RAB9A cooperates with its co-regulatory GTPases to regulate STX13-mediated cargo delivery to mature melanosomes [21].

Herein, we showed the function of circ_0013359 in melanoma growth and glycolysis. In the meantime, we explored the interaction between circ_0013359 and miR-136-5p/member RAS oncogene family (RAB9A) axis in melanoma, which may provide new therapeutic targets for melanoma.

## 2 Materials and methods

### 2.1 Tissue collection

All melanoma specimens (*n* = 30) and adjacent normal tissues (*n* = 30) were obtained from Hebei Chest Hospital. All tissue specimens were stored at −80°C immediately after surgical excision. Some clinical characteristics of melanoma patients are listed in Table 1.

**Table 1:** Correlation between circ_0013359 expression and clinical characteristics in melanoma patients (*n* = 30)

Clinicopathologic parameters	Case	circ_0013359 expression		*P*-value*
		Low (*n* = 15)	High (*n* = 15)	
**Age (years)**				0.4561
≤60	12	5	7	
>60	18	10	8	
**Gender**				0.2690
Male	17	10	7	
Female	13	5	8	
**TNM stage**				0.0080*
I–II	11	9	2	
III	19	6	13	
**Distal metastasis**				0.0253*
No	18	12	6	
Yes	12	3	9	

*P < 0.05.


**Informed consent:** Informed consent was obtained from all individuals included in this study.
**Ethical approval:** The research related to human use has been complied with all the relevant national regulations and institutional policies, and in accordance with the tenets of the Helsinki Declaration and has been approved by the Ethics Committee of Hebei Chest Hospital.

### 2.2 Cell culture

Human melanoma cell lines (A875 and SK-MEL-1) were purchased from Tongpai Biotech (Shanghai, China) and cultured in Dulbecco’s modified Eagle’s medium (DMEM; Hyclone, Logan, UT, USA) containing 10% fetal bovine serum (FBS; Hyclone). Human epidermal melanocytes (HEMn-LP) were commercially obtained from Invitrogen (Carlsbad, CA, USA) and maintained in M254 medium (Invitrogen) containing Human Melanocyte Growth Supplement (Invitrogen). All cells were cultured in an incubator supplemented with 5% CO_2_ at 37°C.

### 2.3 Cell transfection

Small interfering RNA (siRNA) against circ_0013359 (si-circ_0013359; 5′-UGUCCUUUCUCUCCAUAUGCA-3′) and the control (si-NC), miR-136-5p mimics (miR-136-5p) and negative control (miR-NC), miR-136-5p inhibitor (anti-miR-136-5p) and negative control (anti-miR-NC), RAB9A overexpression vector pcDNA3.1-RAB9A (RAB9A) and the control (vector) were synthesized by Genechem (Shanghai, China). These oligonucleotides and vectors were transfected into A875 and SK-MEL-1 cells using Lipofectamine 3000 (Invitrogen) when cell confluence reached ∼80%.

### 2.4 Quantitative reverse transcriptase-polymerase chain reaction (qRT-PCR)

RNA was extracted from tissues and cells using Trizol reagent (Solarbio, Beijing, China). Next, the specific RT-PCR kit (Takara, Dalian, China) was used to synthesize complementary DNA. For RNase R digestion experiment, 2 μg of RNA was treated with or without RNase R (Seebio, Shanghai, China). Then, the RNA expression was measured using SYBR Green PCR Master Mix (Takara) and quantified via the 2^−ΔΔCt^ method. Glyceraldehyde 3-phosphate dehydrogenase or U6 was regarded as an internal control. All primers are presented in Table 2.

**Table 2:** All primer sequences for qRT-PCR

Primer name	Sequence
circ_0013359-F	CCAGGTCCTGTGATAAATGGC
circ_0013359-R	CGACAAGCATACCAGGCTCA
COL11A1-F	ACCCTCGCATTGACCTTCC
COL11A1-R	TTTGTGCAAAATCCCGTTGTTT
miR-136-5p-F	ACACTCCAGCTGGGACTCCATTTGTTTTGAT
miR-136-5p-R	CCAGTGCAGGGTCCGAGGT
RAB9A-F	AGGGACAACGGCGACTATC
RAB9A-R	TCTGACCTATCCTCGGTAGCA
GAPDH-F	GGGAAACTGTGGCGTGAT
GAPDH-R	GAGTGGGTGTCGCTGTTGA
U6-F	CTCGCTTCGGCAGCACA
U6-R	AACGCTTCACGAATTTGCGT

### 2.5 Cell viability assay

Transfected A875 and SK-MEL-1 cells were plated into 96-well plates at a density of 2 × 10^3^ cells/well and incubated at 37°C for 0, 24, 48, or 72 h. Then, the medium with 10 μL 3-(4,5-dimethyl-2-thiazolyl)-2,5-diphenyl-2-*H*-tetrazolium bromide (MTT; 5 mg/mL, Solarbio) was added into each well, followed by incubation for 4 h. Then, dimethyl sulfoxide (Solarbio) was added to dissolve formazan. Finally, optical density (OD) value was tested using a Microplate Reader (BioTek, Burlington, VT, USA).

### 2.6 Colony formation assay

Transfected A875 and SK-MEL-1 cells were injected into 6-well plates at a density of 5 × 10^2^ cells/well and incubated at 37°C for 2 weeks. Later, the cells were stained with crystal violet (Solarbio). Then, the clones were photographed and counted under a microscope.

### 2.7 Flow cytometry

Cell apoptosis was assessed using Annexin V-FITC/propidium iodide (PI) Apoptosis Detection kit (Vazyme, Nanjing, China) following the manufacturer’s instructions. The apoptosis rates of A875 and SK-MEL-1 cells were measured using FACScan Flow Cytometry (BD Biosciences, San Diego, CA, USA).

Transfected A875 and SK-MEL-1 cells were harvested and trypsinized. Subsequently, the pellets were suspended in phosphate-buffered saline (Solarbio). Then, the cells were treated with RNase A (Seebio) and stained with PI (Abcam, Cambridge, UK). Finally, cell phase was monitored using FACScan Flow Cytometry (BD Biosciences).

### 2.8 Transwell assay

Transfected A875 and SK-MEL-1 cells were plated into the upper chamber (Corning, Corning, NY, USA). Meanwhile, the lower chamber was filled with DMEM supplemented with 10% FBS. After culturing for 24 h, the cells were fixed with methanol and stained with crystal violet (Solarbio). Later, the transferred cells were counted under a microscope at 100× magnification. In addition, cell invasion assay was different from cell migration assay in that transwell was pre-coated with Matrigel (Corning).

### 2.9 Detection of glucose consumption and lactate production

Transfected A875 and SK-MEL-1 cells were maintained in 6-well plates. The medium was replaced with fresh medium and incubated for another 48 h. Then, Glucose and Lactate Assay Kits (Abcam) were used to detect glucose consumption and lactate production.

### 2.10 Extracellular acidification rate (ECAR)

The ECAR was detected using the Seahorse XF^e^ 96 Extracellular Flux Analyzer (Seahorse Bioscience, North Billerica, MA, USA). Briefly, cells (2 × 10^4^) were seeded into a Seahorse XF 96 cell culture microplate. Subsequently, glucose, oligomycin, and 2-deoxyglucose were added to each well in sequence at the specified time. All values were normalized to protein concentration. The Seahorse XF-96 Wave software was used to analyze the data. All values were normalized to protein concentration.

### 2.11 Western blot assay

After extracting the protein with RIPA buffer (Solarbio), the equal amount of protein was separated by polyacrylamide gel electrophoresis and transferred onto polyvinylidene fluoride membranes (Corning). Later, the membranes were blocked with 5% skimmed milk and then probed with primary antibodies against hexokinase 2 (HK2; 1:5,000, ab227198; Abcam), lactate dehydrogenase A (LDHA; 1:2,000, ab84716; Abcam), RAB9A (LDHA; 1:2,000, ab235538, Abcam), or GAPDH (1:2,500, ab9485; Abcam). Then, the membranes interacted with a secondary antibody (1:25,000, ab205718; Abcam). The signal intensity was examined using an ECL system (Beyotime, Shanghai, China).

### 2.12 Dual-luciferase reporter assay

Circ_0013359 sequence containing the wild-type or mutant binding site of miR-136-5p was inserted into the 3′-UTR end of the reporter gene of pmirGLO vector (LMAI Bio, Shanghai, China) to construct circ_0013359 WT or circ_0013359 MUT reporter. Meanwhile, RAB9A 3′-UTR harboring miR-136-5p wild-type or mutant binding site was inserted into pmirGLO vector (LMAI Bio) to form RAB9A 3′-UTR WT or RAB9A 3′-UTR MUT reporter. Subsequently, the luciferase reporter and miR-136-5p or miR-NC were introduced into A875 and SK-MEL-1 cells. The luciferase intensity was determined using Dual-Lucy Assay Kit (Solarbio). *Renilla* luciferase activity was used for normalization.

### 2.13 Xenograft assay

A875 cells (1 × 10^6^) stably transfected with sh-NC or sh-circ_0013359 (Genechem) were subcutaneously injected into the right abdomen of 5-week-old BALB/c nude mice (*n* = 5 per group). Subsequently, tumor volume was measured every 7 days. After 35 days, the mice were killed and the xenograft tumors were weighed. The levels of circ_0013359, miR-136-5p, and RAB9A in the excised tumor tissues were measured by qRT-PCR or western blot.


**Ethical approval:** The research related to animal use has been complied with all the relevant national regulations and institutional policies for the care and use of animals and was approved by the Animal Ethics Committee of Hebei Chest Hospital.

### 2.14 Statistical analysis

Data were displayed as mean ± standard deviation using GraphPad Prism 7 software (GraphPad Inc., La Jolla, CA, USA). The differences were evaluated by Student’s *t*-test (two groups) or one-way analysis of variance (multiple groups). The correlation among circ_0013359, miR-136-5p, and RAB9A was tested via Spearman’s correlation coefficient. *P* < 0.05 was considered statistically significant.

## 3 Results

### 3.1 Circ_0013359 expression is increased in melanoma tissues and cells

**Figure 1:**
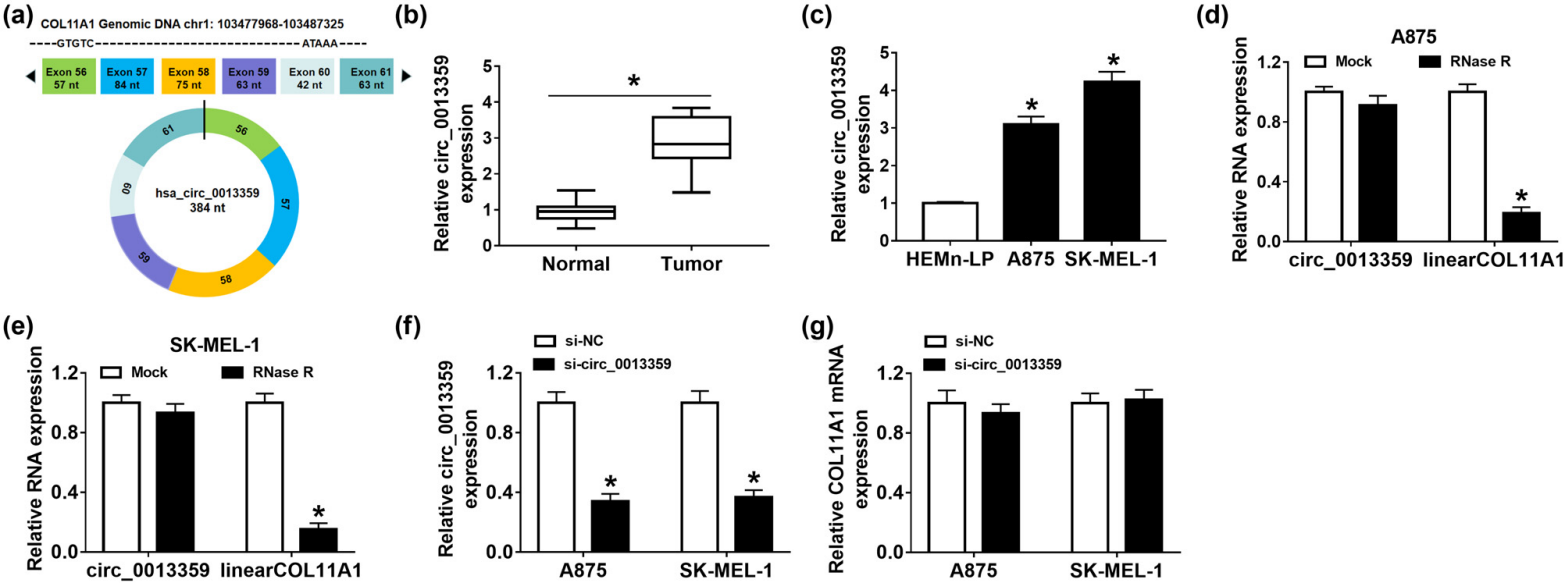
Circ_0013359 expression is increased in melanoma tissues and cells. (a) The schematic diagram showed the cyclization of circ_0013359. (b) Expression of circ_0013359 in melanoma tissues (*n* = 30) and adjacent normal tissues (*n* = 30) was determined by qRT-PCR. (c) Circ_0013359 level was measured in HEMn-LP, A875, and SK-MEL-1 cells. (d and e) After RNase R treatment, qRT-PCR was used to detect the levels of circ_0013359 and linear COL11A1. (f and g) The levels of circ_0013359 and COL11A1 mRNA were detected in A875 and SK-MEL-1 cells transfected with si-NC or si-circ_0013359. *P < 0.05.

First, the schematic diagram displayed that circ_0013359 was derived from exon 57 to exon 63 of COL11 A1 gene (Figure 1a). To investigate the potential role of circ_0013359 in melanoma, we tested the expression of circ_0013359 in melanoma tissues and adjacent healthy tissues. The results suggested that the circ_0013359 level was markedly elevated in melanoma tissues compared to normal tissues (Figure 1b). In addition, we observed that circ_0013359 expression in A875 and SK-MEL-1 cells was strikingly higher than that in HEMn-LP cells (Figure 1c). As illustrated in Figure 1d and e, the expression of linear COL11A1 was significantly reduced after RNase R digestion, while circ_0013359 was resistant to RNase R. Next, the knockdown efficiency of circ_0013359 was detected in A875 and SK-MEL-1 cells, and the results showed that si-circ_0013359 transfection inhibited the expression of circ_0013359 but had no effect on the expression of COL11A1 mRNA (Figure 1f and g). As displayed in Table 1, circ_0013359 expression was not associated with age and gender but was related to TNM stage and distal metastasis. These data revealed that circ_0013359 might play a carcinogenic role in melanoma.

### 3.2 Depletion of circ_0013359 inhibits proliferation, migration, invasion, and glycolysis and induces apoptosis and cycle arrest in melanoma cells

**Figure 2:**
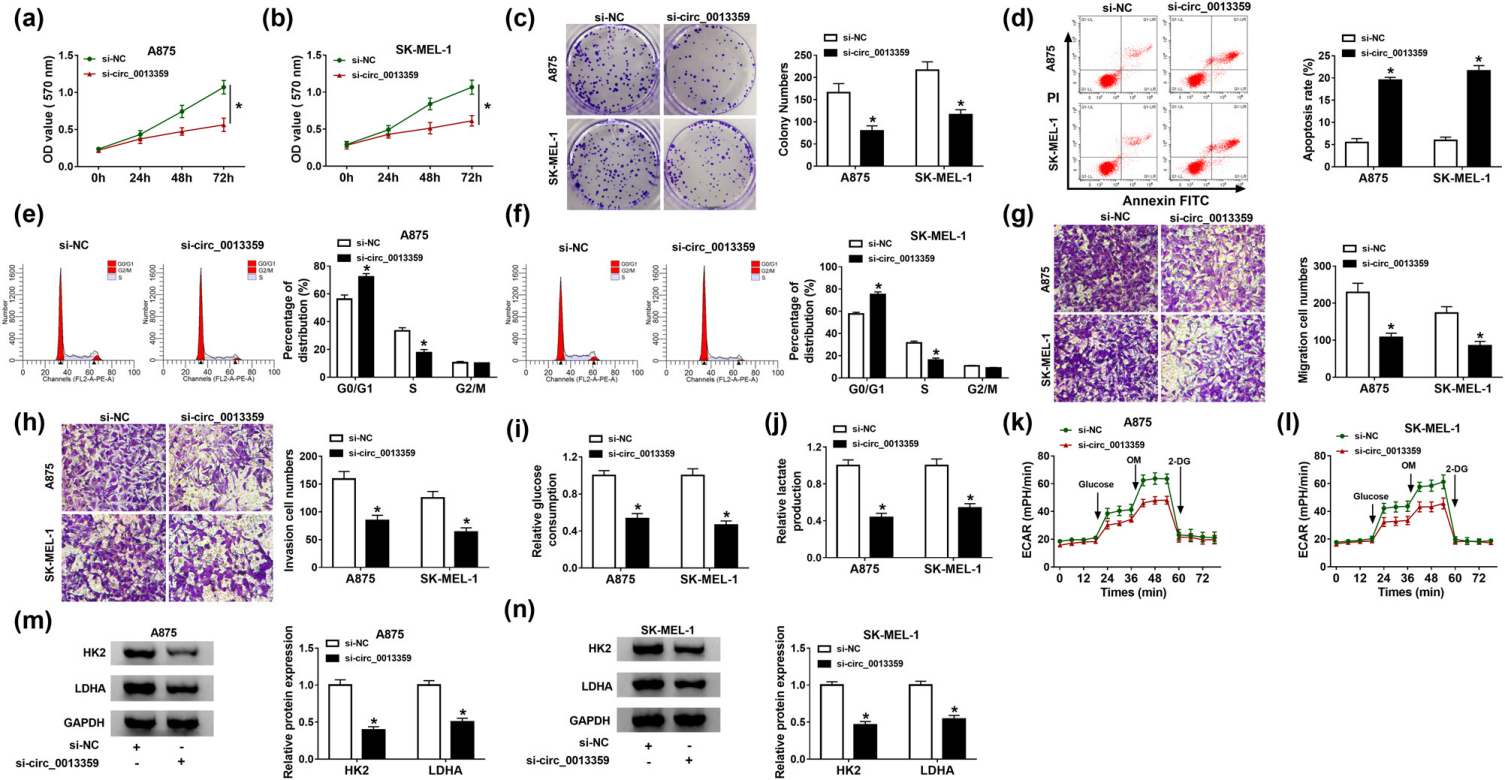
Depletion of circ_0013359 inhibits proliferation, migration, invasion, and glycolysis and induces apoptosis and cycle arrest in melanoma cells. A875 and SK-MEL-1 cells were introduced with si-NC or si-circ_0013359. Cell viability (a and b), colony numbers (c), apoptosis (d), and cell cycle distribution (e and f) were detected by MTT assay, colony formation assay, and flow cytometry, respectively. (g and h) Transwell assay was used to assess cell migration and invasion. (i and j) Glucose consumption and lactate production were measured after transfection for 48 h. (k and l) Seahorse XF^e^ 96 Extracellular Flux Analyzer was used to detect ECAR. (m and n) The levels of HK2 and LDHA were tested using western blot. *P < 0.05.

To explore the biological function of circ_0013359 in melanoma, loss-of-function experiments were conducted by transfecting si-NC or si-circ_0013359 into A875 and SK-MEL-1 cells. MTT analysis revealed that silencing of circ_0013359 prominently inhibited the viability of A875 and SK-MEL-1 cells (Figure 2a and b). In addition, colony formation assay exhibited that the colony number of A875 and SK-MEL-1 cells was significantly decreased in the si-circ_0013359 transfection group (Figure 2c). Flow cytometry showed that circ_0013359 knockdown increased the apoptosis rate of A875 and SK-MEL-1 cells (Figure 2d). Furthermore, circ_0013359 downregulation increased the percentage of cells in the G0/G1 phase and decreased the percentage of cells in the S phase (Figure 2e and f). Transwell assay indicated that introduction of si-circ_0013359 suppressed the migration and invasion of A875 and SK-MEL-1 cells (Figure 2g and h). In addition, glucose consumption and lactate production were remarkably reduced in the si-circ_0013359 group relative to the control group (Figure 2i and j). Besides, knockdown of circ_0013359 strikingly decreased ECAR in comparison with the control group (Figure 2k and l). Moreover, transfection with si-circ_0013359 overtly repressed the protein expression of HK2 and LDHA (Figure 2m and n). Overall, these data evidenced that circ_0013359 depletion restrained the progression of melanoma.

### 3.3 Circ_0013359 directly interacts with miR-136-5p

**Figure 3:**
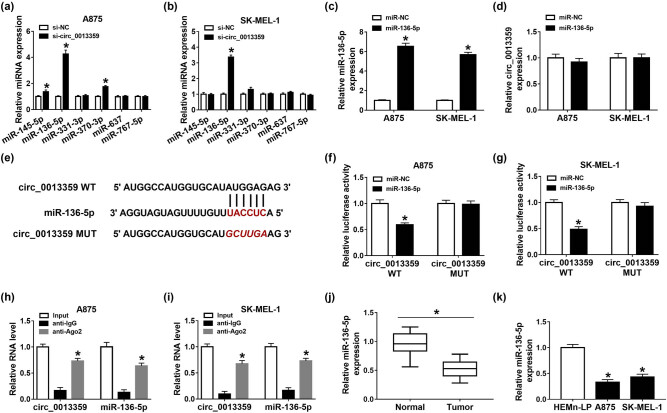
Circ_0013359 directly interacts with miR-136-5p. (a and b) The levels of the selected six targets (miR-145-5p, miR-136-5p, miR-331-3p, miR-370-3p, miR-637, and miR-767-5p) were detected in A875 and SK-MEL-1 cells transfected with si-NC or si-circ_0013359. (c and d) The levels of miR-136-5p and circ_0013359 were measured in A875 and SK-MEL-1 cells transfected with miR-NC or miR-136-5p. (e) The predicted binding site between circ_0013359 and miR-136-5p was exhibited. (f and g) The luciferase activity was tested in A875 and SK-MEL-1 cells co-transfected with circ_0013359 WT or circ_0013359 MUT and miR-NC or miR-136-5p. (h and i) RIP assay was used to confirm the binding relationship between circ_0013359 and miR-136-5p. (j) MiR-136-5p level was detected in melanoma tissues and normal tissues. (k) Expression of miR-136-5p was examined in HEMn-LP, A875, and SK-MEL-1 cells. *P < 0.05.

Next, we used the circinteractome online database to predict the possible targets of circ_0013359 and tested the expression of the selected six targets (miR-145-5p, miR-136-5p, miR-331-3p, miR-370-3p, miR-637, and miR-767-5p) after siRNA-mediated knockdown of circ_0013359. The results showed that the increased level of miR-136-5p was the most significant; therefore, miR-136-5p was selected as the research object (Figure 3a and b). Compared with the control group, transfection with miR-136-5p remarkably increased the miR-136-5p level but had no effect on the circ_0013359 level (Figure 3c and d). As shown in Figure 3e, the potential binding site between circ_0013359 and miR-136-5p was predicted by the circinteractome online database. To verify the relationship between circ_0013359 and miR-136-5p, dual-luciferase reporter assay was performed by co-transfecting circ_0013359 WT or circ_0013359 MUT and miR-NC or miR-136-5p into A875 and SK-MEL-1 cells. The results showed that miR-136-5p mimics prominently reduced the luciferase activity of circ_0013359 WT reporter (Figure 3f and g). Besides, RNA immunoprecipitation (RIP) analysis exhibited that circ_0013359 and miR-136-5p were significantly enriched in the anti-Ago2 group compared with the anti-lgG group (Figure 3h and i). qRT-PCR analysis showed that miR-136-5p expression in melanoma tissues was remarkably decreased compared to normal tissues (Figure 3j). In addition, the miR-136-5p level in A875 and SK-MEL-1 cells was markedly reduced relative to HEMn-LP cells (Figure 3k). Thus, these data demonstrated that circ_0013359 could bind to miR-136-5p.

### 3.4 Circ_0013359 silencing reduces melanoma cell progression and is restored by anti-miR-136-5p treatment

**Figure 4:**
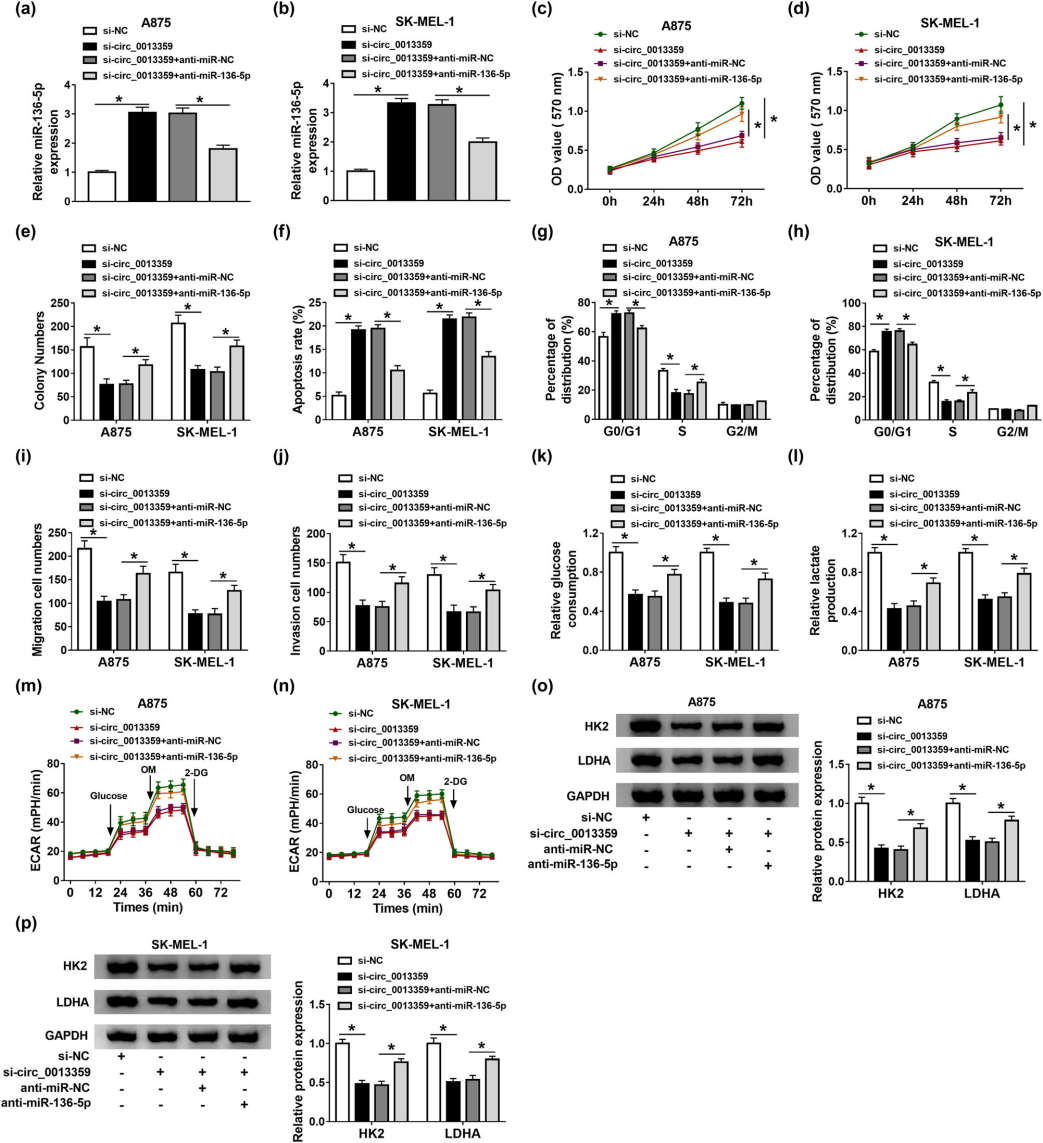
Circ_0013359 silencing reduces melanoma cell progression and is restored by anti-miR-136-5p treatment. A875 and SK-MEL-1 cells were transduced with si-NC, si-circ_0013359, si-circ_0013359 + anti-miR-NC, or si-circ_0013359 + anti-miR-136-5p, respectively. MiR-136-5p level (a and b), cell viability (c and d), colony numbers (e), apoptosis rate (f), cell cycle distribution (g and h), and the number of cell migration and invasion (i and j) were evaluated using appropriate methods. Glycolysis was assessed by measuring glucose consumption (k), lactate production (l), ECAR (m and n), and the protein levels of HK2 and LDHA (o and p). *P < 0.05.

To illuminate whether circ_0013359 regulated melanoma development by targeting miR-136-5p, A875 and SK-MEL-1 cells were divided into four groups: si-NC, si-circ_0013359, si-circ_0013359 + anti-miR-NC, and si-circ_0013359 + anti-miR-136-5p. First, circ_0013359 knockdown strikingly elevated the level of miR-136-5p in A875 and SK-MEL-1 cells, whereas anti-miR-136-5p transfection abolished the increase (Figure 4a and b). MTT and colony formation assays revealed that circ_0013359 silencing restrained melanoma cell proliferation, whereas this impact was abrogated by inhibiting miR-136-5p expression (Figure 4c–e). In addition, depletion of circ_0013359 promoted the apoptosis and cycle arrest of A875 and SK-MEL-1 cells, while these changes were reversed after anti-miR-136-5p transfection (Figure 4f–h). Simultaneously, introduction of anti-miR-136-5p weakened the inhibition of circ_0013359 knockdown on cell migration and invasion (Figure 4i and j). Furthermore, downregulation of circ_0013359 suppressed glycolysis of A875 and SK-MEL-1 cells by reducing glucose consumption, lactate production, ECAR, HK2, and LDHA expression, while these effects were reverted by co-transfecting of si-circ_0013359 and anti-miR-136-5p (Figure 4k–p). To sum up, these data evidenced that circ_0013359 silencing hindered melanoma development by sponging miR-136-5p.

### 3.5 MiR-136-5p directly targets RAB9A

**Figure 5:**
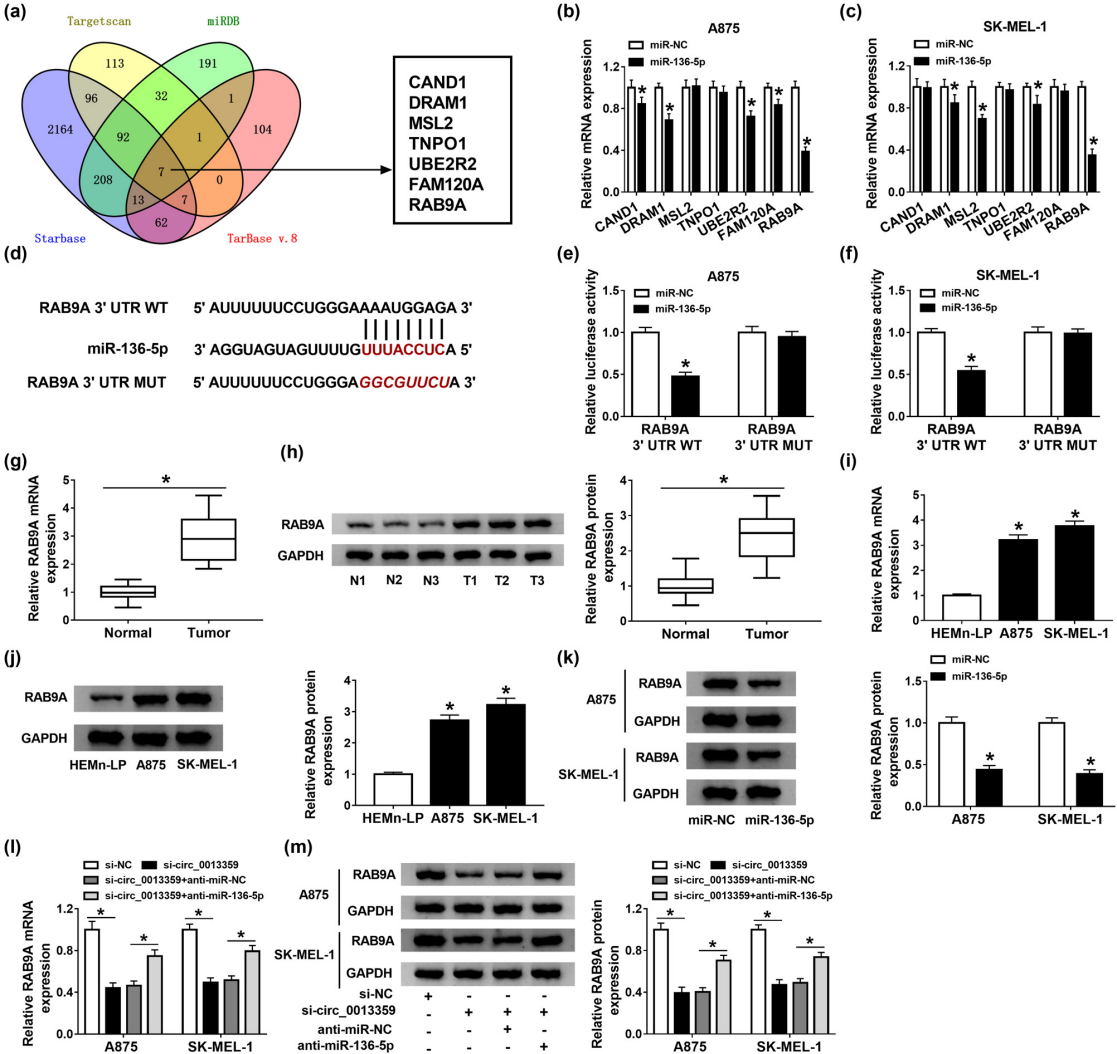
MiR-136-5p directly targets RAB9A. (a) Venn diagram showing seven possible target genes for miR-136-5p in four databases (Starbase, Targetscan, miRDB, and TarBase v.8). (b and c) The expression of seven possible target genes was detected in A875 and SK-MEL-1 cells transfected with miR-NC or miR-136-5p. (d) The putative binding sequence between miR-136-5p and RAB9A 3′-UTR was displayed. (e and f) Dual-luciferase reporter assay was used to verify the relationship between miR-136-5p and RAB9A. (g and h) The mRNA and protein levels of RAB9A were tested in melanoma tissues and normal tissues. (i and j) The expression of RAB9A in HEMn-LP, A875, and SK-MEL-1 cells was measured by qRT-PCR and western blot. (k) RAB9A protein level was determined in A875 and SK-MEL-1 cells transfected with miR-NC or miR-136-5p. (l and m) The mRNA and protein levels of RAB9A were examined in A875 and SK-MEL-1 cells introduced with si-NC, si-circ_0013359, si-circ_0013359 + anti-miR-NC, or si-circ_0013359 + anti-miR-136-5p. *P < 0.05.

Furthermore, the possible target genes of miR-136-5p were hypothesized using Starbase, Targetscan, miRDB, and TarBase v.8 (Figure 5a). Subsequently, the expression of seven target genes shared in four databases was detected using qRT-PCR in A875 and SK-MEL-1 cells transfected with miR-NC or miR-136-5p. The results showed that RAB9A expression was most significantly downregulated after miR-136-5p transfection (Figure 5b and c). As displayed in Figure 5d, miR-136-5p and RAB9A 3′-UTR had putative binding sequences. Then, dual-luciferase reporter assay showed that mature miR-136-5p overtly decreased the luciferase activity of RAB9A 3′-UTR WT reporter (Figure 5e and f). Compared with normal tissues, RAB9A mRNA and protein levels were significantly increased in melanoma tissues (Figure 5g and h). Meanwhile, RAB9A mRNA and protein levels in A875 and SK-MEL-1 cells were remarkably higher than those in HEMn-LP cells (Figure 5i and j). In addition, miR-136-5p overexpression inhibited RAB9A protein expression (Figure 5k). Moreover, the mRNA and protein levels of RAB9A were prominently reduced after circ_0013359 knockdown, whereas introduction of anti-miR-136-5p eliminated the impact (Figure 5l and m). These results indicated that circ_0013359 indirectly regulated RAB9A expression via targeting miR-136-5p.

### 3.6 RAB9A overexpression mitigates the inhibitory effect of miR-136-5p on melanoma cell progression

**Figure 6:**
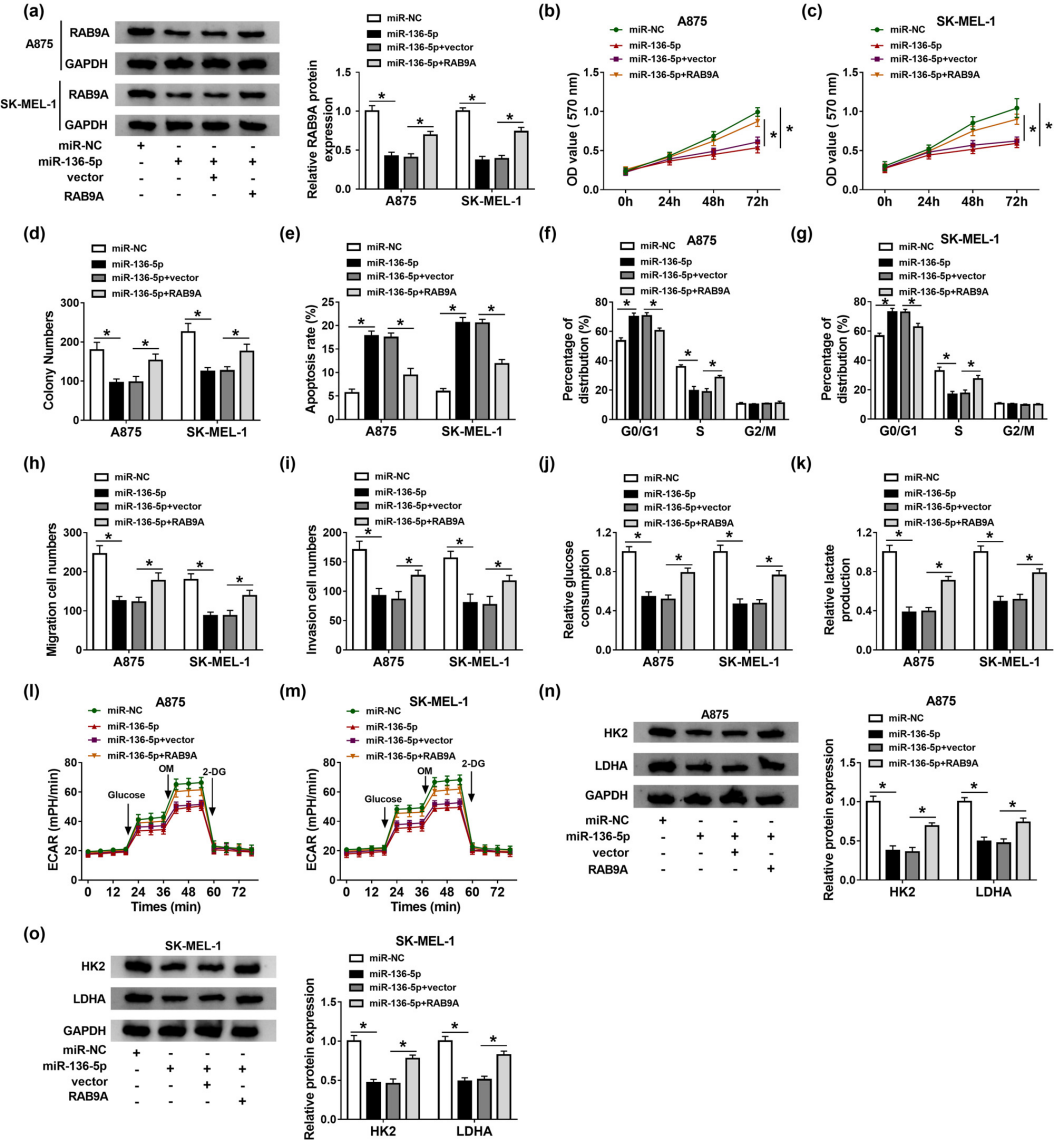
RAB9A overexpression mitigates the inhibitory effect of miR-136-5p on melanoma cell progression. A875 and SK-MEL-1 cells were transfected with miR-NC, miR-136-5p, miR-136-5p + vector, or miR-136-5p + RAB9A, respectively. RAB9A protein level (a), cell viability (b and c), colony numbers (d), apoptosis rate (e), cell cycle progress (f and g), cell migration and invasion (h and i), glucose consumption (j), lactate production (k), ECAR (l and m), and the protein levels of HK2 and LDHA (n and o) were examined via the appropriate methods. *P < 0.05.

To elucidate the association between miR-136-5p and RAB9A in melanoma development, A875 and SK-MEL-1 cells were transfected with miR-NC, miR-136-5p, miR-136-5p + vector, or miR-136-5p + RAB9A. First of all, transfection with RAB9A abolished the reduction in RAB9A protein level caused by miR-136-5p overexpression (Figure 6a). In addition, upregulation of miR-136-5p suppressed proliferation, migration, and invasion and triggered apoptosis and cycle arrest in A875 and SK-MEL-1 cells, while these effects were overturned by overexpressing RAB9A (Figure 6b–i). Moreover, miR-136-5p transfection led to significant decreases in glucose consumption, lactate production, ECAR, HK2, and LDHA levels, while these impacts were abrogated by upregulating RAB9A (Figure 6j–o). These data further validate the inhibitory role of miR-136-5p in melanoma cell progression via targeting RAB9A gene.

### 3.7 Silencing of circ_0013359 inhibits the growth of melanoma xenografts

**Figure 7:**
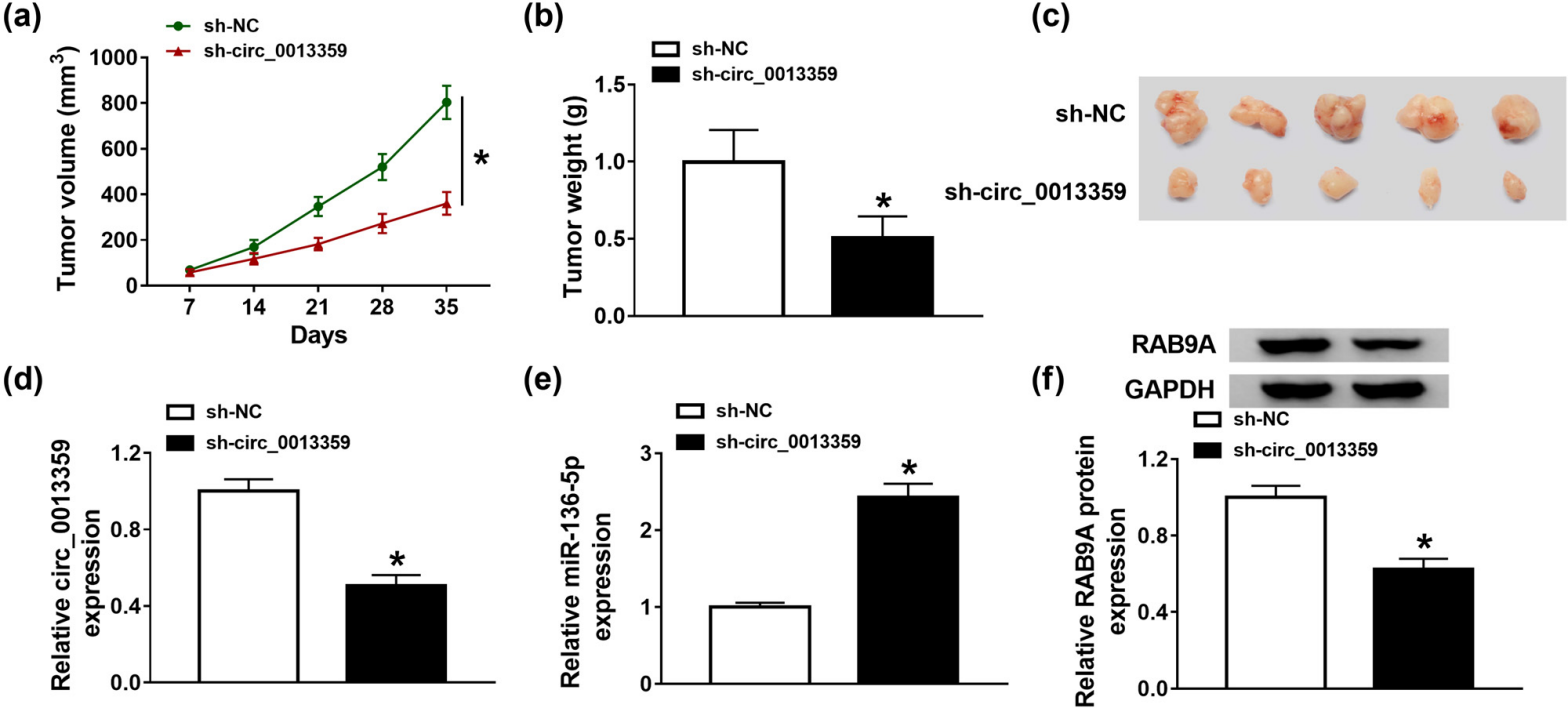
Silencing of circ_0013359 inhibits the growth of melanoma xenografts. A875 cells (1 × 10^6^) stably transfected with sh-NC or sh-circ_0013359 were subcutaneously injected into the nude mice. (a) Tumor volume was measured every 7 days. (b) After the mice were killed, the xenograft tumors were weighed. (c) The photos of tumor on day 35 were shown. (d–f) The levels of circ_0013359, miR-136-5p, and RAB9A were measured by qRT-PCR or western blot. *P < 0.05.

To evaluate the function of circ_0013359 *in vivo*, we established a melanoma xenograft model by transfecting sh-NC or sh-circ_0013359 into A875 cells. As exhibited in Figure 7a, tumor volume was strikingly decreased in the sh-circ_0013359 group compared with that of the sh-NC group. After cell injection for 35 days, tumor weight in the sh-circ_0013359 group was markedly reduced relative to that of the sh-NC group (Figure 7b and c). Besides, the expression of circ_0013359 and RAB9A was restrained, and miR-136-5p expression was promoted in the sh-circ_0013359 group compared to that of the sh-NC group (Figure 7d–f). Overall, these data suggested that knockdown of circ_0013359 blocked melanoma growth *in vivo*.

## 4 Discussion

In the present research, circ_0013359 associated with glycolysis was identified in melanoma. Recently, glucose metabolism reprogramming becomes a new hallmark of tumor cells [22]. HK2 is a rate-limiting enzyme for glycolysis, and its reduction leads to limited glycolysis [23]. LDHA converts pyruvate to lactate to accelerate glycolysis [24]. Besides, ncRNAs exert significant effects on glucose metabolism reprogramming by interacting with key transcription factors or glycolysis-related enzymes [25]. Therefore, exploring the role of circRNAs in glycolysis is helpful to develop new pathways to control abnormal metabolic phenotypes, thereby providing promising targets for tumor therapy. Herein, we verified that the circ_0013359 level was conspicuously increased in melanoma and its reduction repressed melanoma cell glycolysis by reducing glucose consumption, lactate production, ECAR, HK2, and LDHA.

Furthermore, substantial previous studies have corroborated that circRNAs serve as competing endogenous RNAs to compete for miRNA binding sites, thereby regulating a series of biological functions [26]. For example, circ_0020710 expedited the development and immune evasion of melanoma by sponging miR-370-3p to indirectly regulate CXCL12 expression [27]. In addition, circ_0002770 contributed to the progression of melanoma via interacting with miR-331-3p and elevating DUSP5/TGFBR1 expression [28]. Moreover, Zhou et al. discovered that circRNA_0016418 accelerated the growth and mobility of melanoma by absorbing miR-625 to elevate YY1 expression [29]. In this report, silencing of circ_0013359 suppressed melanoma cell proliferation, mobility, and glycolysis.

Moreover, we demonstrated that circ_0013359 directly interacted with miR-136-5p. We chose miR-136-5p as the research object depending on its anti-cancer effect in various cancers, including renal cell carcinoma [30], cervical cancer [31], and lung squamous cell carcinoma [32]. Han et al. suggested that augmentation of miR-136-5p impeded tumor progression by mediating the FAM83H-AS1/miR-136-5p/MTDH pathway in triple-negative breast cancer [33]. Huang et al. disclosed that lncRNA DSCAM-AS1 sponged miR-136-5p to accelerate the malignant phenotypes of melanoma [34]. In this research, the miR-136-5p level was dramatically decreased in melanoma. More importantly, downregulation of circ_0013359 hampered melanoma cell progression via sponging miR-136-5p.

Compelling evidence has revealed that miRNAs contribute to post-transcriptional silencing of target genes by base pairing with 3′-UTR of mRNAs [35]. In the present study, we speculated that RAB9A and miR-136-5p had a binding relationship based on the prediction results of many online software, which was verified by dual-luciferase reporter analysis. Furthermore, Liang et al. revealed that the RAB9A level was strikingly elevated in melanoma, and RAB9A silencing hindered melanoma cell growth [36]. In this report, we clarified that miR-136-5p blocked melanoma cell development via targeting RAB9A.

## 5 Conclusion

In short, these findings unveiled that circ_0013359 could sponge miR-136-5p to regulate RAB9A. Furthermore, circ_0013359 contributed to melanoma progression via modulating miR-136-5p/RAB9A axis, suggesting that circ_0013359 might be a promising biomarker for melanoma treatment.
